# Gut Microbiota Extracellular Vesicles as Signaling Molecules Mediating Host-Microbiota Communications

**DOI:** 10.3390/ijms222313166

**Published:** 2021-12-06

**Authors:** Salma Sultan, Walid Mottawea, JuDong Yeo, Riadh Hammami

**Affiliations:** 1 NuGut Research Platform, Faculty of Health Sciences, School of Nutrition Sciences, University of Ottawa, Ottawa, ON K1N 6N5, Canada; ssult017@uottawa.ca (S.S.); wmott020@uottawa.ca (W.M.); jyeo@uottawa.ca (J.Y.); 2 Department of Microbiology and Immunology, Faculty of Pharmacy, Mansoura University, Mansoura 35516, Egypt; 3 Department of Biochemistry, Microbiology and Immunology, Faculty of Medicine, University of Ottawa, Ottawa, ON K1H 8M5, Canada

**Keywords:** gut microbiota, microbiota extracellular vesicles, molecular signalling, microbiota-host communications, microbial metabolites, gut microbiota-brain axis

## Abstract

Over the past decade, gut microbiota dysbiosis has been linked to many health disorders; however, the detailed mechanism of this correlation remains unclear. Gut microbiota can communicate with the host through immunological or metabolic signalling. Recently, microbiota-released extracellular vesicles (MEVs) have emerged as significant mediators in the intercellular signalling mechanism that could be an integral part of microbiota-host communications. MEVs are small membrane-bound vesicles that encase a broad spectrum of biologically active compounds (i.e., proteins, mRNA, miRNA, DNA, carbohydrates, and lipids), thus mediating the horizontal transfer of their cargo across intra- and intercellular space. In this study, we provide a comprehensive and in-depth discussion of the biogenesis of microbial-derived EVs, their classification and routes of production, as well as their role in inter-bacterial and inter-kingdom signaling.

## 1. Introduction

**Figure 1:**
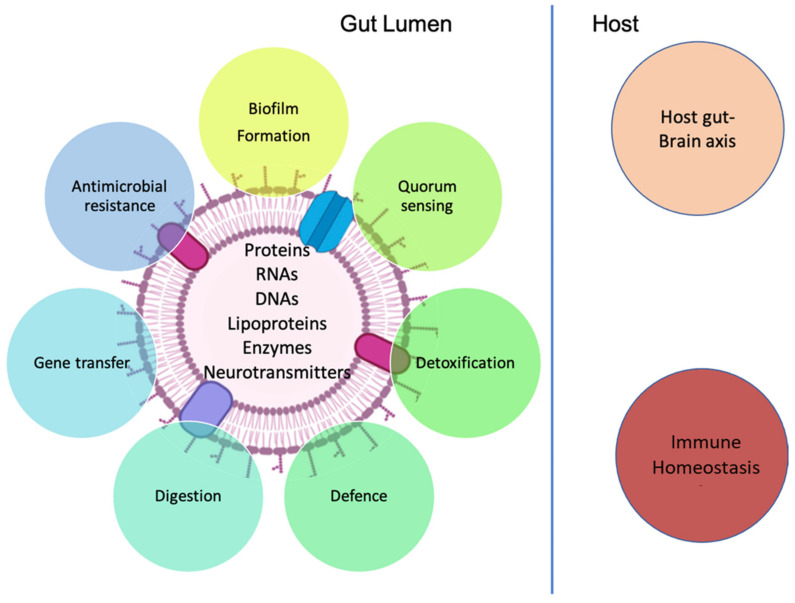
Roles of MEVs in interbacterial and microbiota–host signaling. Microbiota extra vesicles (MEVs) contribute to the communication between gut commensals including transfer of antimicrobial resistance genes [19], horizontal gene transfer [20], biofilm formation [21], quorum sensing [22], detoxification [23], and digestion. Furthermore, MEVs and their cargoes induce immune homeostasis [24,25] and act as a communication approach in the gut–brain axis [26,27].

The gut microbiota is the most significant microbial ecosystem in the human body. Its huge gene content and diversity enable this ensemble to exhibit many beneficial functions to the host, including nutritional, physiological, and immunological roles that collectively contribute to human health [1,2,3]. Host–gut microbiota crosstalk has been extensively reported for multiple health and disease statuses [4,5,6,7,8,9,10,11]. This bidirectional communication is thought to be mediated through metabolic, immunological, endocrine, and neuronal pathways [12]. Recently, a new channel of communication through secreted microbiota extracellular vesicles (MEVs) began to appear. It is commonly believed that the communication between Gram-negative bacteria and the host is mediated by secreted vesicles, known as outer-membrane vesicles (OMVs) [13]. Gram-positive bacteria have also been reported to generate EVs [14]. In 2013, Kang et al. [15] characterized microbiota-derived EVs in mouse stools. They illustrated that stool MEVs from an IBD mouse model exhibited severe dysbiosis compared to the change in the microbiota composition between the inflammation and control phenotypes [15]. While it was not clear whether this dysbiosis was a consequence or a cause of the inflammation, this study illustrates that EVs play a regulatory role in intestinal immunity and homeostasis [15]. For instance, the EVs of the gut microbe *Akkermansia muciniphila* protected mice from developing colitis and lowered the production of the proinflammatory cytokine, IL6, in response to *E. coli* treatment [15]. Additionally, *A. muciniphila* EVs were reported to induce serotonin secretion in both the colon and hippocampus of mice, suggesting MEVs’ potential as signaling molecules in the gut–brain axis [16]. A more recent report has shown that MEVs may cross intestinal barriers and reach distal organs, such as the liver and adipose tissues, inducing insulin resistance and glucose intolerance [17]. EVs derived from *Lactobacillus plantarum* have exhibited an antidepressant-like effect [18]. Collectively, this supports the hypothesis that gut microbiota-derived EVs may act as inter-bacterial and host-microbe signaling pathways that regulate intestinal homeostasis and human health, even in distal organs (Figure 1). In this review, we discuss the biogenesis of microbiota-derived EVs. We focus on the role of microbiota-derived vesicles (MEVs) in inter-bacterial signaling and host–microbiota interactions. This covers only one direction of the communication from the microbiota towards the host. The other direction of the crosstalk will be covered in a future review.

## 2. Gut Microbiota

The gut microbiota refers to a collective complex, dynamic microbial community along the gastrointestinal tract’s length (GIT) that reaches its maximum density at the colon [28]. This ensemble of microbes includes bacteria, viruses, archaea, and eukaryotes [29,30]. The gut microbial gene content was estimated to be 150-fold that of humans, and more than 99% of these genes belong to bacteria [29]. Approximately 1150 bacterial species have been identified in the human gut, with an average of 160 species per individual [29]. Gut bacteria are dominated by the two phyla, Bacteroidetes and Firmicutes, which constitute more than 70% of the gut bacteria with low proportions of phyla like Actinobacteria, Proteobacteria, Fusobacteria, and Verrucomicrobia [31].

Gut microbiota play critical roles in human health. They are a significant factor in shaping and evolving the immune system [1]. They also metabolize indigestible plant fibers to generate essential metabolites, such as short-chain fatty acids (SCFAs) [2]. Major SCFA producers include Clostridial clusters IV and X1Va, *Bacteroides* and *Bifidobacterium* [32]. The major microbiota-generated SCFAs include butyrate, propionate, and acetate, where colonocytes mainly utilize butyrate as the primary energy source while acetate and propionate act as substrates of lipogenesis and gluconeogenesis in peripheral tissues [11,33]. Additionally, the integrity of the intestinal barrier is controlled by SCFAs. For example, butyrate upregulates the expression of tight junction-associated proteins [3]. In addition to the colonic fermentation of dietary fibers, the gut microbiota interacts with other host metabolic processes, such as the regulation of bile acid metabolism, the metabolism of choline, and insulin resistance [2].

Microbiota–host interaction involves not only the host’s sensing of bacterial metabolites, but also direct interaction with the bacteria. This last point is particularly confusing, since most bacteria are physically separated from the host by the mucus layer. Moreover, live bacteria’s effects are often different from those of heat-killed bacteria, suggesting that bacterial membrane components’ recognition is more than just a passive interaction. Shen et al., in 2012, first demonstrated the phenomenon by showing that commensal bacteria produce EVs [34]. They reported that the administration of EVs isolated from *Bacteroides fragilis* simulated similar benefits compared to administering the bacteria itself. This finding, soon followed by others, opened a new perspective from which to understand how gut bacteria affect host homeostasis and, importantly, to understand the systemic and distal impact of gut bacteria on the host. This review discusses our current knowledge regarding the functions of microbiota-derived EVs on the host’s health as a shuttle for transferring bioactive cargoes (i.e., proteins, mRNA, miRNA, DNA, carbohydrates, and lipids), as well as their potential role as signaling pathways (Figure 1).

## 3. Gut Microbiota-Derived Extracellular Vesicles

As a part of the communication process between organisms, the gut microbiota produces small bodies, called microbial extracellular vesicles (MEVs). They carry the message of antibiotics’ resistance to the surrounding bacteria [19,35]. Moreover, they act as an efficient system for the detoxification of components that are unfavorable to bacterial growth [23]. In 2017, Bryant, W.A., suggested that commensal bacteria-derived vesicles could contribute to colonization in the gastrointestinal tract [36].

### 3.1. Biogenesis

**Figure 2:**
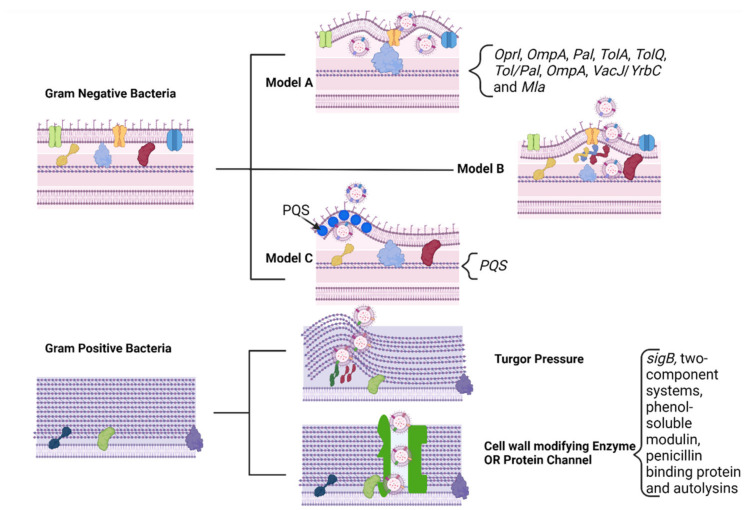
Biogenesis of EVs from both Gram-negative and Gram-positive bacteria. Gram-negative OMVs are produced when the outer membrane asymmetry is achieved (Model A), misfolded proteins are condensed in the outer membrane (Model B), and lipopolysaccharides are modified (Model C) [40]. On the other hand, Gram-positive bacteria may vesiculate following a turgor pressure or via the action of cell-wall-modifying enzymes or protein channels [56]. Figure created with BioRender.com (accessed on 15 October 2021).

Bacteria are categorized into two classes, according to their outer membrane nature: Gram-negative (G−) and Gram-positive (G+) bacteria. G− bacteria are characterized by a double plasma membrane separated by periplasm. Vesicles arising from the outer membrane blebbing of G− bacteria are called Outer-Membrane Vesicles (OMVs) [37]. They carry periplasmic contents, such as lipoproteins, lipids, and outer membrane proteins [38]. Furthermore, some pathogenic G− bacteria produce another type of vesicle, called Inner Outer Membrane Vesicles (IOMVs). They contain pieces from both cytoplasmic and periplasmic membranes and are enriched with ATPs and DNA [39]. Three models demonstrating OMV production were reviewed by C. Volgers and his team in 2018 [40] (Figure 2). These models suggest that the production technique maintains the outer membrane homeostatic state. Accordingly, OMVs are produced when outer membrane asymmetry is achieved (Model A), misfolded proteins are condensed in the outer membrane (Model B), and lipopolysaccharides are modified (Model C). The outer membrane of G− bacteria is characterized by the asymmetric distribution of lipids with lipopolysaccharides on the outer side and phospholipids on the inner side of the membrane [41]. Defects in this asymmetric distribution lead to increased microbial vesiculation (Model A). Furthermore, model A can be achieved by reduced interactions between the outer membrane lipids and the peptidoglycan layer [42]. The genes involved in this model include genes encoding the proteins associated with the peptidoglycan layer, such as: Oprl, OmpA, Pal or TolA in *Pseudomonas aeruginosa* [43]; TolA, TolQ and Tol/Pal in *E. coli* [44,45]; OmpA in *Acinetobacter baumannii* [46]; and the ABC-transporter VacJ/YrbC in *Haemophilus influenzae* and *V. cholerae* [47], and its homolog, Mla, in *E. coli* [41]. The deletion or under-regulation of these genes reduces the interaction between the peptidoglycan and the outer membrane, which grows faster and increases microbial vesiculation [47]. The second model B suggests that the accumulation of misfolded protein or peptidoglycan fragments presses on the outer membrane and results in the protrusion of the membrane and vesicle generation. This can be triggered by temperature stress or defects in cell wall remodeling [47]. The third model is currently specific to *Ps. aeruginosa*. It enriches the membrane’s curvature inducing molecules such as B-band lipopolysaccharide and the quinolone, PQS. PQS is hypothesized to induce anionic repulsion among membrane lipopolysaccharides and form a stable salt bridge between the negatively charged B-band lipopolysaccharide and cationic salts, which results in a membrane curvature and asymmetric expansion of the outer leaflet of the membrane compared to the inner leaflet [48].

Since the cell membranes of G+ bacteria exhibit a different nature, one thick layer of peptidoglycans, they were not considered extracellular vesicle producers until the discovery of MEVs from *Staphylococcus aureus* by Lee EY et al., in 2009 [14]. In addition, the studies by Rivera J. et al. in 2010 and Jeon J. et al. in 2017 reported the production of MEVs by *Bacillus anthracis* and *Cutibacterium acnes* [49,50]. Different mechanisms are utilized by G+ bacteria to release MEVs when compared to G− bacteria (Figure 2): in a sense, they push the vesicles through the thick membrane through turgor pressure, protease lysis, or protein channels [51]. The genetic regulation of the vesiculation in G+ bacteria is established for the general regulators *sigB* and two-component systems [52,53]. The EVs’ formation in *Staphylococcus aureus* relies on phenol-soluble modulins, which are amphipathic alpha-helical peptides that disrupt the cytoplasmic membrane, in addition to a reduction in peptidoglycan cross-linking [54]. The reduction in peptidoglycan crosslinking suggests a role for cell-wall-modifying molecules, such as penicillin binding protein and autolysins, in EVs biogenesis. This is supported by the detection of these molecules in EVs, as revealed by mass spectrometry [55]. Finally, the differences in the phospholipids between EVs and their parental cells indicate that EVs are generated at specific locations [56].

### 3.2. Biomarkers

Extracellular vesicles (EVs) are abundant in all body fluids, including plasma, saliva, urine, semen, cerebral spinal fluid (CSF), bronchial fluid, and breast milk [57]. They can readily cross physiological barriers due to their good stability and small dimensions [58]. This is why they are considered a beneficial source of biomarkers in circulation [59]. Since they are enriched by molecular contents, such as nucleic acids, lipids, and a wide collection of proteins [60,61], MEVs are implicated in many disorders, including neurodegenerative diseases, such as Alzheimer’s disease and Parkinson’s disease [62,63,64]. Combes et al. (2004) demonstrated the correlation between EVs and the occurrence of neurological syndrome-like cerebral malaria [65]. Furthermore, the normalization of these vesicles during the recovery period suggests their potential as biomarkers of disease intensity. More recently, carbohydrase 1 (CA-1) and S100A8 were identified through proteomics analysis as cargoes of EVs in cerebral malaria syndrome. They are specifically increased during pathogenesis, reinforcing the notion of these molecules as biomarkers in malaria [66]. Cancer [67,68,69] and stroke [70] have also been reviewed to show the potential of EVs as biomarkers.

Notably, EVs’ contents are present in different originating forms, either as components of parent cells or membrane-associated particles. During the biogenesis of EVs, different cargoes (i.e., mRNA, DNA, proteins, lipids, etc.) are packed into the vesicles; this could be used as a surrogate indicator of parent cells’ to provision of specific cell-origin biomarkers [71,72].

**Figure 3:**
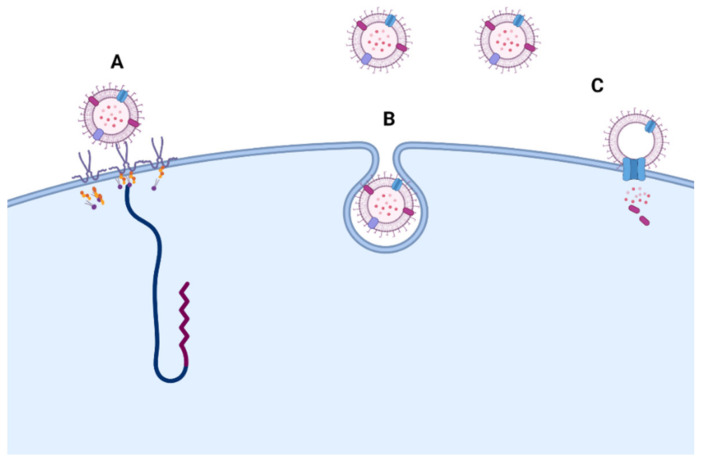
Routes of MEV entry into host cells. MEVs may interact with host cells by either (**A**) binding with the cell receptor and activating a cellular response; (**B**) fully incorporating into the cellular cytoplasm; or (**C**) delivering their content to the host cell [73]. Figure created with BioRender.com (accessed on 15 October 2021).

Three main mechanisms that describe the way in which MEVs interact with their host were presented by O’Donoghue et al., in 2016 [73]: (i) the full in corporation in the host’s cytoplasm; (ii) the activation of the host’s receptors; and (iii) the delivery of their bacterial content (Figure 3). More focus was added to study the activation of the host’s receptors by pathogen-associated molecular pattern (PAMP) induced by pathogenic bacteria other than commensal strains [74].

### 3.3. MEVs and Cellular Communication

#### 3.3.1. Role in Inter-Bacterial Signaling

EVs play a variety of roles in bacterial crosstalk (Table 1). *Haemophilus influenza* generates and receives DNA-containing EVs; EVs play a significant role in transferring DNA among bacteria by protecting it from nucleases [20], thus indicating the deep involvement of EVs in horizontal gene transfer in inter-bacterial communication. EVs released from *Bacteroides* possess β-lactamases that protect gut commensals and pathogens from β-lactam antibiotics [19]. Bacterial EVs also represent a means of detoxifying harmful molecules, including misfolded proteins, toxic materials, and viral particles [23,75,76,77]. Additionally, bacteria EVs play an essential role in bacterial quorum sensing. For example, EVs from *Ps. aeruginosa* contain pseudomonas quinolone signal molecules, enabling *Ps. aeruginosa* to live in nutrient-poor environments [78]. Within the gut lumen, gut microbiota-derived EVs act as delivery vehicles for digestive enzymes, including glycosidases and proteases, that hydrolyze the complex polysaccharides into simple nutrients for other commensals in the gut [79,80].

EVs contribute to the formation of biofilms, such as *H. pylori* and *Ps. aeruginosa* [81,82]. Moreover, EVs could be a valuable means of protecting other/neighbor strains of bacteria by enveloping toxic compounds inside vesicles. For instance, some strains of *Sulfolobus* release EVs containing sulfolobicin toxins that can kill other strains even within the same genus [83]. By contrast, EVs derived from certain bacterial strains possess antimicrobial activities against competitor microbes. Wang et al. recently illustrated that *Burkholderia thailandensis* releases outer membrane vesicles (OMVs) with antimicrobial activities against drug-resistant and competitor microbial species, including methicillin-resistant *Staphylococcus aureus* (MRSA) [84]. A similar antimicrobial effect was reported for OMVs released by *Ps. aeruginosa* [85].

An important role of EVs in molecular exchange between bacterial cells is their role in phage transfer between bacterial cells [86]. The presence of prophages has been shown to induce *S. aureus* vesiculation compared to prophage-devoid cells [87]. Initially, EVs were viewed as antiphage protectors due to their lowering of the phage concentration through adsorption. For instance, the efficiency of T4 bacteriophage infection was reduced by binding to OMVs of *E. coli* [88]. The same study revealed the role of OMVs in innate bacterial defense by neutralizing antimicrobial peptides [88]. Additionally, EVs released by marine Cyanobacteria have been shown to defend marine bacteria against phage infection through the sequestration of phages by EVs containing the phage receptors [89]. By contrast, a more recent study illustrated that bacterial extra vesicles promote phage infection in phage-resistant bacteria by sharing surface components, including phage receptors or attachment molecules from phage sensitive cells to phage resistant cells [90]. Furthermore, bacteriophages were capable of injecting their genetic materials in minicells that resemble EVs [91], indicating that EVs may facilitate the transfer of phage genetic materials between cells.

#### 3.3.2. Role in Inter-Kingdom Signaling

Bacteria-derived EVs, especially those from gut microbiota, can cross eucaryotic cell membranes and intestinal cell walls [92]. Microbiota-derived vesicles can be phagocytosed by immune cells of lamina propria [34], and they can be detected in blood and urine [93]. DNA of bacterial origin has been detected in the serum of healthy subjects, which is known as DNAemia [94]. Indeed, bacterial DNA originating from bacteria membrane vesicles was found in plasma [93]. This finding implies that microbiota-generated vesicles can penetrate different barriers, such as the intestinal epithelium and the vascular endothelium, to reach distant locations inside the host. Two distinct pathways have been suggested for bacterial vesicles to cross the intestinal wall; the paracellular and transcellular pathways [92]. EVs can alter the composition of the tight junction through which they may enable the parental pathogen to invade the intestinal epithelium. For example, vesicles from *Campylobacter jejuni* break down the junction proteins E-cadherin and occludin to enable *C. jejuni* invasion [95]. On the other hand, vesicles from commensal bacteria increase the expression of tight junction proteins to limit paracellular transport [96]. Furthermore, the probiotic *Escherichia coli* Nissle 1917 strain generates outer membrane vesicles that regulate the expression of tight junction proteins, ZO-1 and ZO-2, in the intestinal epithelium cells [97]. Bacteria-generated vesicles can also enter the host cells through the endocytic pathway, as reviewed in O’Donoghue and Krachler [73]. It has been shown that bacterial outer membrane vesicles utilize the four types of endocytosis to invade the host cells, including clathrin-mediated, actin-dependent, caveolin-mediated, or clathrin-caveolin-independent endocytosis [73].

### 3.4. MEVs and Immune Homeostasis

Gut microbiota-derived EVs (MEVs) play a significant role in maintaining gut immune homeostasis (Table 2). MEVs enclose multiple copies of microorganism-associated molecular patterns, including periplasmic proteins, DNA, RNA, LPS and peptidoglycan, which interacts with pattern recognition receptors such as NOD1 and NOD2 and Toll-Like Receptors (TLR) on immune cells to start a cascade of immune signaling [24,25,98,99]. This EV–immune cell interaction relies on the EVs’ cargo, which varies according to the virulence of the source strain. For instance, proteomic analyses have illustrated that only EVs from virulent mycobacterium strains carry the TLR 2 lipoprotein agonist [100]. Additionally, this TLR–EV interaction is selective for the receptor. EVs released by *Lactobacillus* and *Bifidobacterium* genera were found to exert differential effects on TLRs, where they enhanced the cellular responses of TLR 2/1 and TLR 4 while suppressing the responses of TLR 2/6, with no effect on TLR5 [101]. Furthermore, EVs could suppress the immune system through their sRNA and miRNA content; this is the case of sRNA, from the fungus Botrytis cinerea, which suppress plant immunity through gene silencing [102]. Moreover, microRNA (miRNA) generated by anopheline mosquitoes my interfere with the host miRNA and regulate some immune responses [103], indicating that pathogens may utilize EVs as a means of suppressing the host immune system [104].

Commensals-derived MEVs have been shown to regulate gut immune homeostasis. EVs released by *Bacteroides fragilis* have induced the secretion of anti-inflammatory cytokines while reducing the secretion of proinflammatory cytokines [105]. Additionally, they have mediated regulatory Treg responses, which suppressed the mucosal inflammation in a DSS model of colitis [34]. Likewise, the MEVs from *Lactobacillus rhamnosus* induced the expression of IL-10 and enhanced Treg responses in mouse mesenteric lymph nodes and Peyer’s patches [26]. Similarly, Kang et al. [15] reported an important shift in stool MEV composition in a DSS mouse model of IBD compared to controls. In addition, EVs derived from *Akkermansia muciniphila* have been reported to reduce body weight loss, increase colon length, improve epithelial stability, and reduce inflammatory cell infiltration to the colon wall of DSS-treated mice [15]. The same study reported an inverse relationship between the severity of colitis and *A. muciniphila* EVs [15]. Together, this indicates that gut microbiota-derived vesicles play a potential role in maintaining gut immune homeostasis.

### 3.5. MEVs and the Gut-Brain Axis

**Figure 4:**
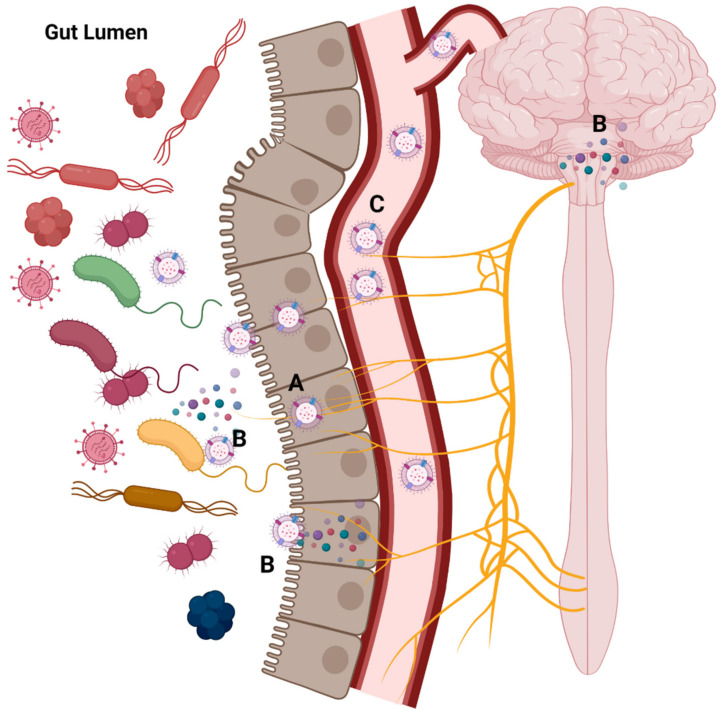
Microbiota-generated extracellular vesicles (MEVs) and gut–brain axis communication. MEVs facilitate gut–brain axis communication through three hypothesized pathways: A—vagal nerve stimulation [26,121]; B—endocrine release modulation from gut bacteria, enterocytes, and hippocampal neurons [16,18]; or C—delivery of cargoes to the CNS through the blood circulation [27]. Figure created with BioRender.com (accessed on 15 October 2021).

The contribution of the microbiota–gut–brain axis to the host’s mental health and neural development has received increasing attention over the past decade. The term microbiota–gut–brain axis refers to the interactions between the gut microbiota and the central nervous system (CNS) through the neural, endocrine, and immune signalling pathways [106]. Sudo et al. [107] reported that germ-free mice possess a hyperactive hypothalamus-pituitary (HPA) axis with a noticeable level of stress-associated hormones compared to mice with conventional microbiota. Various studies showed that the gut microbiota play a critical role in the modulation of anxiety [108,109,110] and memory processing [111]. Diversity in the gut microbiota has been linked to behavioral disorders. At the same time, exposure to non-pathogenic bacteria can harmonize adult animals’ behaviors [108] and anxiety symptoms in human subjects [112,113]. Additionally, CNS development is directly related to exposure to certain commensal bacteria in early life [114,115,116,117]. Although many studies support the microbiota–gut–brain axis’s existence, there is a limited understating of how signals are transferred from the gut to the brain. However, there is evidence that the gut can modulate the CNS through some pathways (Table 3, Figure 4): (i) the gut microbiota can captivate the neural signaling between the brain and the gut through the interaction between the vagal nerve and the enteric nervous system (ENS) [118,119,120,121,122]; (ii) the endocrine response of the host can communicate the gut microbes’ signal to the brain through circulation [123,124]; (iii) the gut microbe can modulate the central and peripheral immune cells, resulting in changes in stress and behavioral responses [125,126,127,128,129,130]; and (iv) gut microbes release metabolites, such as neurotransmitters, that can travel through the circulation of the CNS [131,132].

The recent report on increased levels of systemic LPS-positive bacterial extracellular vesicles in patients with intestinal barrier dysfunction provides some evidence on the capacity of MEVs to circulate systemically [133] and deliver and elicit a variety of immunological and metabolic responses in different organs, including the brain. Recently, gut microbiota-generated MEVs were shown to correlate with the inhibition of energy metabolism in the hypothalamus of MDD patients [134]. Another recent study demonstrated that MEVs derived from *Lactobacillus plantarum* induced antidepressant-like behavior in mice [18], which supports the potential use of MEVs as biotherapeutics in MDD. Al-Nedawi et al. illustrated that EVs from *Lactobacillus rhamnosus* can stimulate the afferent neurons of the enteric nervous system [26]. *L. rhamnosus* is known to spike the vagus nerve, which is an essential signalling pathway in the gut–brain axis [121]. Other investigators have illustrated that EVs from the gut member, *Paenalcaligenes hominis*, cause vagus nerve-dependent cognitive impairment that is reduced by vagotomy [135]. Recently, EVs from *Akkermansia muciniphila* have been reported to induce the secretion of serotonin in mice colon and hippocampus, and in the Caco-2 cell line [16]. Altogether, this supports the hypothesis that MEVs are signaling molecules that could control brain activities.

In addition to being a signaling molecule in the enteric nervous system, MEVs have been demonstrated as cargoes that package psychoactive molecules and shuttle them to distant locations from the gut. Analysis of the EVs released by *Bacteroides fragilis* has revealed their content of histamine and gamma-amino-butyric acid (GABA), the two neurotransmitters that could affect brain functions [27]. RNA in MEVs could also mediate gut brain communications. An assessment of the bacteria RNA content in post-mortem brains of patients with Alzheimer’s illustrated the prevalence of RNA related to Proteobacteria, Firmicutes, *Staphylococcaceae*, *Corynebacteriaceae*, and *Propionibacteriaceae* [136]. Actinobacteria and Firmicutes dominated Alzheimer’s brains along with the depletion of Proteobacteria and Bacteroidetes compared to controls [136].

**Table 3:** Roles of MEVs in microbiota gut–brain axis communications.

Activity	Evidence	Refs
Vagal nerve stimulation	EVs of *Lactobacillus rhamnosus* can stimulate the afferent neurons of the enteric nervous system	[26,121]
	EVs of *Paenalcaligenes hominis*, cause vagus nerve-dependent cognitive impairment	[135]
Endocrine modulation	EVs from *Akkermansia muciniphila* have been reported to induce the secretion of serotonin in mouse colons and hippocampus, and in the Caco-2 cell line	[16]
	Extracellular vesicles derived from *Lactobacillus plantarum* increase brain-d erived neurotrophic factor (BDNF) expression in cultured hippocampal neurons and produce antidepressant-like effects in mice	[18]
Cargoes carrier	EVs released by *Bacteroides fragilis* include histamine and gamma-amino-butyric acid (GABA) as part of their content	[27]
	Patients with Alzheimer’s exhibited a prevalence of RNA related to Proteobacteria, Firmicutes, *Staphylococcaceae*, *Corynebacteriaceae*, and *Propionibacteriaceae* in their brains	[136]

## 4. Conclusions and Perspectives

Accumulating evidence suggests the role of MEVs as signaling molecules that mediate microbiota–host communications. MEVs are representatives of their parental microbes in many communicative activities. In contrast to their microbial origins, they have more accessibility to blood circulation, and they can shuttle their contents to distant locations from the gut, such as the brain. In contrast to individual metabolites and secreted proteins (secretome), MEVs’ contents are enclosed in a bilayer membrane that protects them from lytic enzymes and RNases in the extracellular environment [26] and facilitates their diffusion to distant organs [17]. Still, MEVs are underestimated as a form of communication with the host. Previous studies have focused on the characterization of their proteomic and/or RNA contents or on investigating the correlation of EVs from a specific microbe with specific body responses [26,27,121,135,136,137]. This may be attributed to a lack of standard methods for the isolation and identification of MEV contents, as well as to a lack of well-defined biomarkers isolated from MEVs. Additionally, current methods do not separate host EVs from MEVs. Recently, some approaches have been described to separate bacterial EVs from human body fluids through the implementation of ultrafiltration, density gradient centrifugation, and size exclusion chromatography [133]. Another obstacle is the lack of a reliable method with which to identify the mother bacterial origin of different MEVs or their identified content in a heterogenous microbial community, such as the gut microbiota [138]. Future research is required to illustrate how the variability of the parent microbiome correlates with the variability of MEV contents and production. Furthermore, additional research is required to assess how MEVs are packaged by microbial cells, why these specific molecules are packed, whether they are targeted to specific cells, how they are targeted to host cells, how they release their cargoes, and whether they can cross biological barriers, such as the intestinal barrier and the blood–brain barrier. Despite the several hurdles that must be overcome for the potential exploitation of MEVs as a drug delivery platform for biologics to targeted body locations, the recent developments discussed in this review offer a taste of their emerging role as mediators of host-microbiota interplay.

## Figures and Tables

**Table 1:** Roles of bacteria EVs in inter-bacterial signaling.

Activity	Example Source Organism(s)	Example Affected Organism(s)	Reference
Horizontal gene transfer	*Haemophilus influenza*	*Haemophilus influenza*	[20]
Antimicrobial resistance	*Bacteroides* spp. and *Haemophilus influenza* (β-lactamases)	Gut microbiota Group A streptococci	[19,35]
Detoxification of harmful molecules and stress relief	*E. coli*, *Salmonella enterica* serovar Typhimurium		[23,75,76,77]
Quorum sensing	*Ps. aeruginosa*	*Ps. aeruginosa*	[22,78]
Digestive enzyme carrier	Gut microbiota	Gut microbiota	
Bacterial biofilm	*H. pylori* and *Ps. aeruginosa*	*H. pylori* and *Ps. aeruginosa*	[21,81,82]
Carrier of antimicrobial materials (survival)	*Sulfolobus* spp. *Burkholderia thailandensis*	Same species or drug-resistant and competitor species, including MRSA	[22,83,84,85]

**Table 2:** Evidence and summary of MEVs contribution to maintaining gut immune homeostasis.

Model System/Host Organism	Microbial Species	Experimental Setup/Clinical Context	MEV Gene/Proteins/Lipids Involved	Reference
Mice/epithelial cells	*Helicobacter pylori*, *Pseudomonas aeruginosa* and *Neisseria gonorrhoea*	Measurement of immune responses and antibody production	Peptidoglycan within OMVs	[24]
Human umbilical endothelial cells	Non-pathogenic or pathogenic *E. coli*	Adhesion protein synthesis, cytokine production and necrosis factor (NF)-κB translocation.	OMVs	[99]
Caco-2, HCT-8, and HT-29 intestinal epithelial cell lines	Enterohemorrhagic *Escherichia coli* O157	Interleukin 8 production and Toll-like receptors TLR4, TLR5 and the nuclear factor (NF-κB) activation.	H7 flagellin, cytolethal distending toxin V and O157 lipopolysaccharide (LPS).	[98]
Mice/airway epithelial cells, THP-1-monocytes and -macrophages	Dust EVs	Measuring lung neutrophilic infiltration and inflammation markers, such as IL-8, IL-6, ICAM-1, proIL-1β and TNF-α levels.	EVs	[25]
Mice/alveolar	Mycobacteria	Proteomic analyses of EVs, H&E staining/confocal fluorescence microscopy and flow cytometry.	TLR2 lipoprotein agonists	[100]
Human-derived dendritic cells, THP-1 Blue-CD14 and HEK293 cell lines	Lactobacilli and Bifidobacterium species	Bacterial phagocytosis, bacterial aggregation, and induction of TLRs pathways	Serum-derived EVs	[101]
Human intestinal epithelial cells (Caco-2)	*Bacteroides fragilis*	Toll-lLike receptor 2, Toll-like receptor 4 gene expression (qRT-PCR) and pro-inflammatory (IFNᵧ) and anti-inflammatory (IL-4 and IL-10) cytokines concentration (ELISA)	Isolated OMVs	[105]
Mice/ex vivo model of peristalsis/in situ patch-clamped enteric neurons	*Lactobacillus rhamnosus* JB-1	Proteomic analyses (EVs), flow cytometry, intracellular cytokine staining in presence and absence of receptor inhibitors.	Isolated EVs	[26]
Dextran sulfate sodium (DSS)-treated C57BL/6 mice and colon epithelial cells induced by *Escherichia coli* EV	Gut microbiota and *A. muciniphila*-derived EV	Metagenome sequencing and measuring weight loss, colon length, inflammatory cell infiltration of colon wall and cytokines level.	Isolated EVs	[15]
